# An Opioid-Sparing Strategy for Laparoscopic Sleeve Gastrectomy: A Retrospective Matched Case-Controlled Study in China

**DOI:** 10.3389/fphar.2022.879831

**Published:** 2022-06-14

**Authors:** Yuanyuan Ma, Di Zhou, Yu Fan, Shengjin Ge

**Affiliations:** Department of Anesthesia, Zhongshan Hospital, Fudan University, Shanghai, China

**Keywords:** opioid-sparing, strategy, laparoscopic sleeve gastrectomy, postoperative hospital length of stay, anesthesia

## Abstract

**Background:** Opioid-sparing anesthesia may enhance postoperative recovery by reducing opioid-related side effects. The present study was to evaluate the effect of an opioid-sparing strategy in bariatric surgery.

**Methods:** This study was conducted as a retrospective matched case-controlled (1:1) study. A total of 44 patients receiving either an opioid-based approach (OBA group) or an opioid-sparing strategy (OSA group) who under laparoscopic sleeve gastrectomy were included between May 2017 and October 2020. The primary outcome was the postoperative hospital length of stay (PLOS). Secondary outcomes were the hospital costs, operative opioid consumption, time to recovery, postoperative pain score at rest and rescue antiemetic administered in the PACU.

**Results:** The clinical demographic and operative data in both groups were comparable. There were no significant differences between the two groups in the PLOS (OSA vs. OBA: 6.18 ± 0.23 days vs. 6.73 ± 0.39 days, *p* = 0.24). Compared to the OBA group, opioid consumption in the OSA group was significantly decreased (48.79 ± 4.85 OMEs vs. 10.57 ± 0.77 OMEs, *p* < 0.001). There were no significant differences in the hospital costs, time to recovery, and rescue antiemetic administered, the incidence of intravenous opioids and vasopressor use in the PACU.

**Conclusion:** The opioid-sparing anesthesia for laparoscopic sleeve gastrectomy was feasible but did not decrease the PLOS.

## Introduction

According to the World Health Organization, China has become the country with the largest number of obese people in the world (World Health Organization). Obesity is often associated with multiple diseases, including type 2 diabetes, hypertension and gastroesophageal reflux disease, which increase the risk of cardiovascular accidents and affect the quality of life (Apovian, 2016). Bariatric surgery is an effective method to treat obesity and related diseases (Phillips and Shikora, 2018). With the rapid development of bariatric and metabolic surgery, more than 10,000 cases have been carried out in China by 2019.The surgical methods mainly include laparoscopic sleeve gastrectomy (LSG) and laparoscopic Roux-en-Y gastric bypass (LRYGB). In our center, bariatric surgery primarily performed laparoscopic sleeve gastrectomy.

Enhanced recovery after surgery (ERAS) was first proposed by a Danish surgeon, Henrik Kehlet in 1995 for colonic resections (Bardram et al., 1995). ERAS pathways with evidence-based interventions could enhance recovery, reduce postoperative complications and shorten hospital stays to improving patient prognosis (Ljungqvist et al., 2017). A key principle of ERAS is multimodal analgesia (MMA) during the perioperative period to minimize the use of opioids, provide the best analgesic effect, and prevent opioid-related side effects (Thorell et al., 2016; McEvoy et al., 2017).

Opioids are the main analgesics for general anesthesia, but they have a variety of side effects include respiratory depression, constipation and ileus, urinary retention and sedation (Colvin et al., 2019). Furthermore, opioids can lead to post-operative nausea and vomiting (PONV) incidence increased (Roberts et al., 2005). Based on the above, “opioid-sparing (OS)” and “opioid-free anesthesia (OFA)” were proposed (Beloeil, 2019; Gabriel et al., 2019). Non-opioid analgesic drugs could been used in OS and OFA, including gabapentenoids, non-steroidal anti-inflammatory drugs, ketamine, intravenous lidocaine and α_2_ adrenergic receptor agonists (dexmedetomidine, clonidine) (Bakan et al., 2015; Hontoir et al., 2016; Gabriel et al., 2019; Beloeil et al., 2021).

OS and OFA have been successfully carried out in radical mastectomy and laparoscopic surgery (Hontoir et al., 2016; Devine et al., 2020). This study was to evaluate the safety effectiveness of an opioid-sparing strategy for laparoscopic sleeve gastrectomy (LSG) in our center to optimize its ERAS strategy.

## Patients and Methods

### Patients and Ethics

This observational retrospective study was conducted in a single regional hospital in China, and approval was obtained from the Ethics Committee of Zhongshan Hospital, Fudan University. The main inclusion criteria was age 18 or over. Exclusion criteria included bradycardia or history of chronic use of opioids. From May 2017 and October 2020, a total of 22 patients were enrolled in the OSA group who were underwent laparoscopic sleeve gastrectomy using an opioid-sparing strategy. After 1-to-1 matching, all 22 patients were matched with 22 patients in the OBA group using an opioid-based strategy. Data was extracted from electronic medical records. Consent was obtained from all patients to allow retrospective data analysis without patient identification.

### Anesthesia Protocol

All patients were treated under an ERAS pathway as our described previously (Ma et al., 2021). In brief, patients did not receive any premedication, and were placed in optimum sniffing position. Electrocardiogram, invasive blood pressure (radial artery) and pulse oximetry were monitored routinely. All patients received propofol (1–2 mg/kg), lidocaine (1.5 mg/kg), and rocuronium (0.6 mg/kg) for induction; rocuronium and desflurane (MAC of 0.8–1.0) for maintenance. In the OBA group, opioids (sufentanil, oxycodone or hydromorphone) could be selected according to the judgment of the anesthesiologist to achieve optimal analgesia. In the OSA group, bilateral laparoscopic transversus abdominis plane (TAP) block and rectus sheath block (RSB) were performed by injecting 40 ml of 0.25% ropivacaine after endotracheal intubation. An intravenous bolus of dexmedetomidine (1 μg/kg) and magnesium sulfate (50 mg/kg) was given over 10min. Anesthesia was maintained with a continuous infusion of dexmedetomidine (0.3 μg/kg/h), magnesium sulfate (10 mg/kg/h) and lidocaine (2 mg/kg/h). Additionally, low-dose oxycodone could be used for analgesia. In both groups, parecoxib 40 mg and propacetamol 2 g were administered intravenously for MMA when not contraindicated; tropisetron was given as routine antiemetic prophylaxis. Vasopressors were administered to maintain the mean arterial pressure within ±20% of baseline measurements until closure of the surgical incision.

### Data Collection

We collected characteristics (age, sex, height, weight, BMI), intraoperative and postoperative data of the patients. The primary outcome was the postoperative hospital length of stay (PLOS). Secondary outcomes included intraoperative opioid consumption (converted to oral morphine equivalents, OMEs), time to recovery (defined as the interval from the PACU to SICU or ward), rescue antiemetic administered, intravenous opioids use, the vasopressor use (urapidil or esmolol) and the level of postoperative pain at rest (measured using the VAS Score) in the post-anesthesia care unit (PACU), and the hospital costs.

### Statistical Analysis

Data analysis was performed using SPSS Statistics 24.0. Continuous variables are expressed as mean ± SEM. Categorical variables are presented as number (percentage of patients). As a pragmatic study, we did not calculate a sample size requirement to show a significant difference in the primary outcome. Unpaired *t*-tests were used to compare the continuous variables. Mann–Whitney *U*-test was performed to compare non-normally distributed data. Chi-square test or Fisher’s exact test was used to compare categorical data. The significance level was defined as a two-sided *p*-value < 0.05.

## Results

### Patient Characteristics

There were 223 medical records between May 2017 and October 2020. The opioid-sparing strategy started with a small number of patients, depending on the attending anesthesiologist. A total of 44 patients were matched and analyzed: OSA group (n = 22) and OBA group (n = 22). Clinical characteristics of patients were comparable in both groups including age, height, weight, BMI, sex, ASA score and comorbidity (Table 1).

**TABLE 1:** Baseline patient characteristics.

Variables	OSA group (n = 22)	OBA group (n = 22)	*p*-Value
Age (years)	33.68 ± 2.26	32.77 ± 2.11	0.77
Height (cm)	168.48 ± 1.86	169.22 ± 1.60	0.76
Weight (kg)	116.17 ± 4.38	124.26 ± 4.73	0.22
BMI (kg/m2)	40.81 ± 1.19	43.26 ± 1.41	0.19
Sex (male/female)	10/12	10/12	-
ASA Score (n, %)			0.75
1	8 (36.4%)	7 (31.8%)	
2	14 (63.6%)	15 (68.2%)	
Comorbidity (n, %)			
Hypertension	9 (40.9%)	13 (59.1%)	0.23
Type2 Diabetes	8 (36.4%)	3 (13.6%)	0.16

### Intraoperative Data

Total mean perioperative opioid consumption was significantly higher in the OBA group compared to the OSA group: 48.79 ± 4.85 OMEs vs. 10.57 ± 0.77 OMEs (*p* < 0.001). The operative data of the two groups were comparable in terms of duration of surgery, total fluids administration and urine volume (Table 2). A total of 21 and 17 patients in the OSA group and OBA group were extubated in the operating room without difference, respectively (*p* = 1.00). Three patients with VAS score of 4 received intravenous opioids in the PACU (1 in the OSA group, 2 in the OBA group), and the VAS scores of the others were ≤3. However, the pain scores were not significantly different in the two groups (*p* = 0.54).

**TABLE 2:** Intraoperative data of patients.

Variables	OSA group (n = 22)	OBA group (n = 22)	*p*-Value
Duration of surgery (min)	140.82 ± 5.83	148.45 ± 5.88	0.36
Total fluids administration (ml)	1454.55 ± 57.63	1600.00 ± 52.22	0.07
Urine volume (ml)	191.82 ± 36.48	185.91 ± 33.18	0.91
Opioid consumption (OMEs)	10.57 ± 0.77	48.79 ± 4.85	<0.001
Extubation in OR (n, %)	21 (95.5%)	17 (77.3%)	1.00

OMEs: oral morphine equivalents; OR: operating room.

### Postoperative Data

One patients in the OSA group and two patients in the OBA group (4.5% vs. 9.1%) suffered from clinically important postoperative nausea and vomiting (PONV) and required rescue antiemetic (droperidol) in the PACU. Two patients in the OSA group and four patients in the OBA group (9.1% vs.18.2%) used vasopressor. And this difference was not statistically significant (Table 3). None of patients had perioperative airway adverse events and respiratory complications.

**TABLE 3:** Postoperative data of patients.

Variables	OSA group (n = 22)	OBA group (n = 22)	*p*-Value
In the PACU			
Time to recovery (min)	65.23 ± 2.52	73.41 ± 7.00	0.28
Extubation (n,%)	1 (4.5%)	5 (22.7%)	1.00
Pain score (VAS)			0.54
1	2 (9.1%)	2 (9.1%)	
2	10 (45.5%)	13 (59.1%)	
3	9 (40.9%)	5 (22.7%)	
4	1 (4.5%)	2 (9.1%)	
Intravenous opioids (n,%)	1 (4.5%)	2 (9.1%)	0.61
Rescue antiemetic (n,%)	1 (4.5%)	2 (9.1%)	0.61
Vasopressor use (n,%)	2 (9.1%)	4 (18.2%)	0.67
PLOS (day)	6.18 ± 0.23	6.73 ± 0.39	0.24
Hospital costs (Yuan)	66211.67 ± 1086.76	62401.42 ± 2635.81	0.19

PACU: post-anesthesia care unit; PLOS: postoperative hospital length of stay.

The median PLOS was 0.55 days shorter for patients who were in the OSA group (6.18 ± 0.23 days) compared to the OBA group (6.73 ± 0.39 days). However, this was not a statistically significant difference (*p* = 0.24). The time to recovery and hospital costs were not significantly different between the two groups (65.23 min vs. 73.41 min, *p* = 0.28; 66211.67 Yuan vs. 62401.42 Yuan, *p* = 0.19).

## Discussion

Although gaining prominence in the bariatric surgery, the feasibility and effectiveness of opioid-sparing or opioid-free anesthesia is not yet in evidence in China. This retrospective cohort study was conducted to verify whether an opioid-sparing strategy for LSG could provide adequate anesthesia and analgesia while minimizing opioid consumption. This study suggested that OSA was associated with lower opioid consumption, without negative effects on postoperative pain scores, PONV or PLOS.

OFA has been reported in the thoracic, cardiac and orthopedic surgery (Chin and Lewis, 19762019; Aguerreche et al., 2021; Selim et al., 2021). It has several advantages in bariatric surgery due to the characteristics of obese patients. There are two compelling reasons to reduce opioids in obese patients: 1. A large proportion of obese patients has obstructive sleep apnea or obesity hypoventilation syndrome; 2. Obese patients are more sensitive to opioids. Therefore, they are more likely to occur the upper airway obstruction, postoperative airway adverse events and respiratory complications (Frey and Pilcher, 2003; Troop, 2016; Subramani et al., 2017). However, this study found no perioperative airway adverse events in the both groups.

Multimodal anesthesia is an essential component of ERAS. We conducted an opioid-sparing strategy based on the concept of MMA by combining alpha-2 agonists (dexmedetomidine), lidocaine, magnesium sulfate, anti-inflammatory drugs (parecoxib and propacetamol) and nerve block. A systematic review and meta-analysis based on 23 randomized controlled trials showed that opioid-inclusive anesthesia does not reduce postoperative pain (Frauenknecht et al., 2019). Consistent with this result, we found that the postoperative pain score in the PACU did not differ between groups, indicating that OSA and OBA provided comparable analgesia. Moreover, none of the patients in the OSA group needed conversion to OBA, and hemodynamics assessed by vasopressor requirements were equivalent in the PACU. Dexmedetomidine is an excellent alternative to opioid-free anesthesia due to an anesthetic-sparing effect (Tsaousi et al., 2018). Massoth C and others found that the incidence of postoperative sedation was almost 4-times higher in patients with OFA associated with dexmedetomidine usage (Massoth et al., 2021). In contrast, we found the time to recovery did not differ between the OSA and OBA. However, ketamine was not in our protocol because it was not available in our department. Ketamine has been an option for OFA due to its several advantages. Firstly, it exhibits analgesic and anti-hyperalgesia properties (Suzuki, 2009). Moreover, it could attenuate opioid-induced hyperalgesia and decrease opioid tolerance (Minville et al., 2010). Therefore, the combination of ketamine and dexmedetomidine might improve hemodynamic stability and decrease the need for opioids.

The incidence of PONV after bariatric surgery is relatively high. Previous studies suggested that opioid-free management decreases the incidence of PONV (Ziemann-Gimmel et al., 2014; Salomé et al., 2021). In contrast, a recent randomized controlled study, opioid-free anesthesia did not decrease the incidence of PONV after gynecological laparoscopy (Massoth et al., 2021). Consistent with their results, there was no significant difference of antiemetic requirements in the PACU between the two groups.

Although PLOS was not statistically different in this cohort study, we suggest that use of OSA represents a valuable opportunity to limit unnecessary opioid exposure for obese patients undergoing LSG. There still has several limitations. Firstly, it was a small matched study that was not powered to detect significant differences in PLOS between OSA and OBA. Secondly, the postoperative opioid consumption and symptoms of PONV in both groups were not available due to the retrospective nature of the study. Nevertheless, the present study confirmed the feasibility of OSA in laparoscopic sleeve gastrectomy. We believe further prospective randomized controlled trials are needed to verify the impact of OSA on postoperative nausea, vomiting and pain in the future.

## Conclusion

The present study demonstrated that opioid-sparing anesthesia, including dexmedetomidine, magnesium, lidocaine and regional block for laparoscopic sleeve gastrectomy, was feasible. OSA did not adversely affect surgical duration, postoperative pain, time to recovery, or PLOS.

## Data Availability

The data that support the findings of this study are available from the corresponding author upon reasonable request.
